# Deaths of people with intellectual disabilities: Analysis of deaths in England from COVID‐19 and other causes

**DOI:** 10.1111/jar.12914

**Published:** 2021-07-16

**Authors:** Pauline Heslop, Victoria Byrne, Rachel Calkin, Avon Huxor, Annie Sadoo, Brian Sullivan

**Affiliations:** ^1^ Norah Fry Centre for Disability Studies, School for Policy Studies University of Bristol Bristol UK; ^2^ Computer Science, College of Engineering, Mathematics and Physical Sciences University of Exeter Exeter UK; ^3^ Population Health Sciences, Bristol Medical School University of Bristol Bristol UK

**Keywords:** COVID‐19, intellectual disabilities, mortality

## Abstract

**Background:**

People with intellectual disabilities experience significant health inequities. The aim of this report is to understand the circumstances leading to death from COVID‐19 in people with intellectual disabilities.

**Method:**

Local areas in England prioritised reviewing 200 deaths of adults with intellectual disabilities. Of these, approximately 80% were required to be deaths from suspected or confirmed COVID‐19 as this was the focus of the study; the remainder from other causes. All deaths occurred between 2 March2020 and 9 June 2020.

**Results:**

People with intellectual disabilities differed from the general population in their symptoms of COVID‐19 and age at death. The overall quality of care was rated similar to other deaths of people with intellectual disabilities. Concerns were raised relating to recognising acute deterioration and do not attempt cardio‐pulmonary resuscitation decisions.

**Conclusions:**

Service improvements are indicated in the ways in which people with intellectual disabilities encounter COVID‐19 and experience the disease.

## 1 BACKGROUND

The COVID‐19 pandemic has lacked uniformity in its impact. Some groups are at higher risk of contracting the disease than others, including pregnant women (Phoswa & Khaliq, 2020), people with cancer (Al‐Shamsi et al., 2020) and those already affected by health disparities in relation to age, race, ethnicity, language, income and living conditions (Rozenfeld et al., 2020). The prognosis of the disease is affected by co‐morbidities that include hypertension, diabetes, cardiovascular disease and respiratory diseases. Being male, aged 65 years and older and a smoker affords greater risk of a critical condition from the virus (Zheng et al., 2020).

Pre‐pandemic, people with intellectual disabilities were already known to experience significant health inequities (Emerson & Hatton, 2013; Hosking et al., 2016) and to have high rates of co‐morbidities (Cooper et al., 2018; Heslop et al., 2020; Perera et al., 2020) including respiratory disease (O'Leary et al., 2018), cardiovascular disease (Glover et al., 2017), obesity (Biswas et al., 2010) and epilepsy (Heslop et al., 2020). The COVID‐19 pandemic has heightened concerns that disabled people in general (United Nations, 2020; World Health Organization, 2020) and people with intellectual disabilities in particular (Hatton, 2020; Joint Committee on Human Rights, 2020) would face disproportionately worse impacts of the virus than others.

The learning [intellectual] disabilities mortality review (LeDeR) programme for England was established in 2015 to support local areas to review the circumstances leading to deaths of people with intellectual disabilities aged 4 years and older.^1^ The LeDeR programme is a service improvement initiative, which suggests recommendations for service improvement at national and local levels. It is not a research study per se, and although there is a standard LeDeR methodology, local interpretation of the way in which LeDeR reviews are conducted is commonplace, and the information collected is not the same in each review. In July 2020, the LeDeR programme published a short report describing key information relating to the circumstances leading to death for the first 50 completed LeDeR reviews of those whose deaths had been attributed to COVID‐19.^2^ It suggested that the circumstances leading to deaths of people with intellectual disabilities who had COVID‐19 might be different from the experiences of others in relation to signs and symptoms of the disease, treatment options and quality of care provided and attitudes towards care and treatment.

Given the lack of social studies on the experiences encountered by socially disadvantaged groups during the COVID‐19 pandemic (Siu, 2020), this report builds on the small study of 50 individuals, by further describing the circumstances leading to death from COVID‐19 for a larger number of adults with intellectual disabilities. The aim of this report is to highlight those aspects of the condition itself, or the care provided to those who have died that may have been problematic for people with intellectual disabilities, so that potential inequalities experienced by this population can be addressed. The objectives are as follows:

- Describe the symptoms, presentation and experience of COVID‐19 in a sample of people with intellectual disabilities.
- Extract any learning for future service provision in relation to COVID‐19 in people with intellectual disabilities.

## 2 METHOD

### 2.1 Data source

In England, deaths of people with intellectual disabilities are reported to the LeDeR programme online or by telephone. Anyone can report the death. At notification, key demographic information is collected about the person who has died, as well as their assumed cause of death. The notification details are transferred to the geographical area in which the person lived, and a reviewer is allocated to check and correct the notification details and to review the death using the standard LeDeR methodology. For some deaths, the review process can take many months, depending on the availability of information, the complexity of review required, and the time available to the reviewer. Given the urgency of the situation in which the circumstances leading to deaths of people with intellectual disabilities needed to be better understood, in June and July 2020, local areas across England were asked to prioritise completing reviews of deaths of adults with intellectual disabilities who had died with confirmed or suspected COVID‐19 and whose deaths had been notified to the LeDeR programme.

### 2.2 Case selection

The total number of deaths to be prioritised for review was determined by the number of reviews that NHS England estimated could be completed within a 4‐week time frame given the additional pressures on staff during the COVID‐19 pandemic and the sensitivities of approaching bereaved families so close to the death of their loved one. This was estimated by NHS England to be 200, approximately one in four of all deaths notified during the 100‐day period between 2 March2020 and 9 June 2020.

Of the 200 deaths, approximately 80% were required to be deaths from suspected or confirmed COVID‐19 as this was the focus of the study. Community testing for COVID‐19 was not widely available in England during Spring 2020, so deaths that were suspected to be from COVID‐19 (i.e., reported to have been from COVID‐19 but a test for the virus had not been done) were included in the sample along with those for whom COVID‐19 had been confirmed. The latter were predominantly people who had died in hospital where testing was more widely available.

The remainder (approximately 20% of the deaths) were required to be from other causes and to be able to provide limited comparison with people with intellectual disabilities who had died in the same time period but whose deaths were not from confirmed or suspected COVID‐19.

A sampling frame was developed to ensure that the 200 deaths were representative of geographical region (the seven NHS England regions of England) and demographic characteristics (age group, gender, ethnic group and place of death). Due to the limited number of deaths able to be reviewed, further purposive sampling was not undertaken (e.g., genetic conditions such as Down's syndrome associated with intellectual disabilities). For the adults who had died from COVID‐19, the sampling proportions were based on the characteristics of those who had died from suspected or confirmed COVID‐19 from March to June 2020. For the adults who had died from other causes, the sampling proportions were based on the characteristics of people with intellectual disabilities notified to the programme in 2019. If there were insufficient deaths in a region to meet the minimum sampling threshold in any of the sampling categories (e.g., only 10% of deaths were among 18 to 49‐year‐olds) then all deaths in the category were included.

### 2.3 Analysis

Each of the selected deaths was reviewed using the established LeDeR programme methodology^3^ supplemented with a prompt sheet designed specifically for the review of deaths due to COVID‐19 (Table 1).

**TABLE 1:** Sampling framework

Geographical region	40 from each of the four NHSE regions that had experienced the most deaths of people with intellectual disabilities from COVID‐19 (London, Midlands, North West, South East); 40 spread evenly between the other three NHSE regions combined (East of England, North East and Yorkshire, South West)
COVID‐19 diagnosis	80% with a confirmed or suspected diagnosis of COVID19 at the time of their death; 20% randomly selected from people without a confirmed or suspected diagnosis of COVID‐19
Age group	20% aged 18–49 years; 80% aged 50 years and older
Gender	60% male, 40% female
Ethnic group	80% white British; 20% other ethnic groups
Place of death	60% hospital; 40% other

Completed reviews were sent to the team at University of Bristol for analysis. Quantitative data was collated and analysed using the IBM SPSS software platform as is usual for LeDeR data. Qualitative data was coded using NVivo software using the regular codebook for the programme. Data from the prompt sheets designed specifically for the review of deaths due to COVID‐19 was collated and analysed using Excel spreadsheets, having been linked to key demographic and quality of care variables in the SPSS and NVivo databases.

A combination of a priori codes (developed before the analysis based on previous findings about deaths of people with intellectual disabilities and risk factors and deaths of people in the general population from COVID‐19) and inductive codes (developed whilst examining the data) were used in the analysis. Coding was checked by two or more researchers. There was little disagreement as to how the codes were ascribed, and any differences in interpretation were agreed following discussion.

## 3 RESULTS

From 2 March 2020 to 9 June 2020, 1347 deaths of adults with intellectual disabilities were notified to the LeDeR programme. Of these, 615 (46%) deaths were from suspected or confirmed COVID‐19; 732 (54%) were from other causes.

Those included in our analysis and who are the focus of this paper are 163 deaths from COVID‐19, 27% of the total number of COVID‐19 deaths occurring during this period, and 43 deaths attributed to other causes, 6% of the total number of deaths from other causes occurring during this period.

Table 2 shows the demographic information about those included in this analysis, with additional data to determine how closely the characteristics of those who died from COVID‐19 matched those of other deaths notified to the LeDeR programme.

**TABLE 2:** Demographic information about those included in the analysis, and how closely they match other deaths notified to LeDeR

	206 deaths included in this analysis		Adult deaths notified to LeDeR in 2019	Adult deaths notified to LeDeR with confirmed or suspected COVID‐19(died 2March–9 June 2020)
	Deaths from confirmed or suspected COVID‐19	Deaths from other causes		
Gender				
Male	56%	63%	57%	59%
Female	44%	37%	43%	41%
*Total number* ^a^	*163*	*43*	*2708*	*612*
Age group				
18–49	21%	21%	23%	17%
50–69	48%	49%	49%	51%
70–84	27%	30%	25%	27%
85 and over	4%	0%	3%	5%
*Total number* ^a^	*161*	*43*	*2708*	*615*
Ethnicity				
White (any)	90%	88%	94%	87%
Asian/Asian British	7%	7%	3%	6%
Black African/Caribbean/Black British	1%	5%	2%	4%
Multiple/mixed or other groups	1%	0%	2%	3%
*Total number* ^a^	*155*	*42*	*2576*	*575*
Level of intellectual disabilities				
Mild/moderate	64%	68%	66%	65%
Severe/profound and multiple	36%	32%	34%	35%
*Total number* ^b^	*154*	*41*	*1952*	*324*
Usual place of residence				
Own or family home	18%	30%	26%	21%
Supported living	25%	21%	27%	28%
Residential home	35%	33%	30%	30%
Nursing home	19%	9%	15%	19%
Other	4%	7%	2%	3%
*Total number* ^b^	*163*	*43*	*2098*	*342*

*Note*: Due to rounding, percentages may not total 100%.

^a^ Total number of notifications for which this information is available.

^b^ The information is collected as part of the review process, rather than at notification of the death, so the number relates to completed reviews only.

The group of 163 deaths from COVID‐19 is not significantly different in age group, gender, ethnicity, level of intellectual disabilities or usual place of residence compared to adult deaths notified to LeDeR with confirmed or suspected COVID‐19 and who died during the same time period. A striking difference between COVID‐19 deaths in the LeDeR sample and COVID‐19 deaths in the general population is in relation to age at death. ONS data for the general population of England and Wales reports that 47% of deaths from COVID‐19 were in people aged 85 years and older (ONS, 2020); LeDeR data suggests that just 4% of people with intellectual disabilities who died from COVID‐19 were aged 85 years and older.

### 3.1 Pre‐existing health conditions

All (100%, *n* = 206) of those included in the sample had at least one long‐term health condition. Table 3 shows the most frequently reported long‐term conditions.

**TABLE 3:** The most commonly reported long‐term health conditions (ordered by prevalence in COVID‐19 people) of those included in the sample

Long term condition	COVID‐19		Other causes of death		Total	
	People with this condition (No.)	People with this condition (%)	People with this condition (No.)	People with this condition (%)	People with this condition (No.)	People with this condition (%)
Mobility impairment	121	74	32	74	153	74
Respiratory conditions	117	72	26	60	143	69
Incontinence	101	62	31	72	132	64
Skin conditions	99	61	32	74	131	64
Mental health needs	96	59	25	58	121	59
Constipation	90	55	24	56	114	55
Sensory impairment	90	55	28	65	118	57
Epilepsy	78	48	25	58	103	50
Cardiovascular disease	56	34	12	28	68	33
Hypertension	54	33	9	21	63	31
Falls	53	33	20	47	73	35
Obesity	53	33	9	21	62	30
Gastric reflux	51	31	17	40	68	33
Dental problems	47	29	15	35	62	30
Hand use impairment	40	25	9	21	49	24
Swallowing issues/dysphagia	32	20	12	28	44	21
Dementia	31	19	10	23	41	20
Diabetes	29	18	5	12	34	17
Osteoporosis	29	18	6	14	35	17
Kidney problems	24	15	4	9	28	14
Cerebral palsy	21	13	4	9	25	12

There are no statistically significant differences in the type of long‐term conditions between those who died from COVID‐19 and those who died from other causes. However, as Table 3 shows, there was a trend for adults who died from COVID‐19 to be more frequently reported to have respiratory conditions (72%), compared to those who died from other conditions (60%). Other conditions more frequently reported in people who died from COVID‐19 compared to those who died from other causes were hypertension (33% compared to 21%) and obesity (33% compared to 21%).

### 3.2 Preventative measures to reduce COVID‐19 infections

Some of the key measures to reduce the spread of COVID‐19 in England were for the clinically extremely vulnerable to ‘shield’ themselves from the possibility of catching the virus; for social distancing measures to be put in place and for the use of personal protective equipment (PPE) (HM Government, 2020).

People identified as at high risk of complications from COVID‐19 received a letter from their GP, hospital or other health provider advising them to shield themselves from the virus from the beginning of April 2020. Their name was also held in a central list of ‘clinically extremely vulnerable’ patients. Those shielding were informed that they should stay at home at all times and avoid all face‐to‐face contact for a period of at least 12 weeks. Of the 163 people who died with COVID‐19, 67 (41%) were reported to not be shielding and 17% (*n* = 27) to have been shielded. Twenty of the 27 who were shielding but who died with COVID‐19 lived in a setting with external paid carers—seven lived in a nursing home, seven in a residential care home and six in supported living settings. Information about shielding was not provided for 69 of the people in the study.

Social distancing measures were introduced to minimise social interaction between people and reduce the transmission of COVID‐19. Those who died with COVID‐19 appeared to have similar experiences to those who died from other causes with regards to social distancing. Social distancing measures were problematic for some, particularly if they were reliant on others for moving and handling, were supported by a number of different paid carers, or lived in small‐sized rooms.

PPE was mentioned in almost half of the reviews. Generally, reviewers found PPE was available and being used by staff. This was the case for those who died with COVID‐19 and who died from other causes. Where there were issues with PPE this was in relation to procurement, the impact that seeing staff in facemasks had on those receiving care and support, and confusion about government guidelines.

### 3.3 The likely source of COVID‐19 infections

For 79 people who died from confirmed or suspected COVID‐19, the likely source of infection was other residents or staff in their care home (52%), a recent hospital stay (27%) or a source in the community (18%). For the other 89 people who died from COVID‐19, the likely source of the infection was unknown.

### 3.4 Symptoms of illness

The key symptoms of COVID‐19 in the general population are a high temperature, a new, continuous cough, and/or a loss of, or change to, the sense of smell or taste. NHS England reports that most people with COVID‐19 have at least one of these symptoms (NHS, 2020).

In our sample of 163 deaths of people with intellectual disabilities from COVID‐19, a wide range of symptoms were reported. These are summarised in Table 4.

**TABLE 4:** The most commonly reported symptoms of illness in those who died from COVID‐19 and those who died from other causes

Symptom	COVID‐19‐related deaths		Deaths from other causes	
	Number	%	Number	%
Difficulty breathing	127	78	14	33
Cough/‘chesty’	104	64	13	30
Fever	93	57	5	12
One of the above symptoms only	35	21	14	33
Two of the above symptoms	53	33	6	14
All three of the above symptoms	61	37	2	5
Recent urine or chest infection	64	39	17	40
Lethargy/tiredness	64	39	10	23
Generally unwell	54	33	15	35
Loss of appetite	49	30	13	30
Diarrhoea or vomiting	33	20	9	21
Confusion	16	10	3	7
Sore throat	5	3	2	5
Abdominal pain	5	3	7	16
Loss of sense of smell or taste	0	—	0	—
Other symptoms	32	20	14	33
No symptoms	0	—	3	7

The most frequently reported symptoms of illness in those who died from COVID‐19 were difficulty breathing (78%), a cough (64%) or fever (57%). These symptoms were significantly more frequently reported in people who died from COVID‐19 than in people who died from other causes (Chi‐square corrected for multiple comparisons *p* < .001 for each symptom). Of those who died from COVID‐19, 37% had all three symptoms; 39% had two of the symptoms and 21% had one of these symptoms.

None of those who died from COVID‐19 were reported to have had a loss of sense of smell or taste, although this is a key symptom in people in the general population. There was a non‐significant trend for lethargy and tiredness to be more frequently reported in people who died from COVID‐19 (39%) compared with other causes (23%).

### 3.5 Access to healthcare

Access to healthcare has come under scrutiny during the COVID‐19 pandemic and the use of NHS111 online and NHS111's role in responding to calls about COVID‐19 added a potentially additional layer of complexity for people with intellectual disabilities. In addition, the *COVID‐19 rapid guideline: critical care in adults* published by NICE in March 2020 recommended the use of a frailty index, which disadvantaged people with intellectual disabilities from accessing critical care (NICE, 2020). The guideline was changed in April 2020 to clarify that the index ought not be used with people with intellectual disabilities. We were, therefore, interested if reviewers reported any problems with accessing healthcare for the people with intellectual disabilities in the sample.

A total of 28% (*n* = 45) of the 163 completed reviews of people who died from COVID‐19, and 30% (*n* = 13) of the 43 completed reviews of deaths from other causes, noted problems that a person had in accessing timely and appropriate healthcare.

For people who died from COVID‐19, problems with access to healthcare were varied and included NHS111 service calls not being returned or returned later than scheduled leading in some cases to an emergency 999 call being used. Access to testing for COVID‐19 was also referred to several times in completed reviews, and requests for COVID‐19 testing in care homes were reported as being declined or unavailable. Support from specialist intellectual disability nurses in acute hospitals was also discussed as problematic by several reviewers.

For people who died from other causes, many references to access to healthcare were in relation to access before the pandemic, including it not being known that a person had intellectual disabilities, and the person not attending or being offered annual health checks or other appropriate services. There were also a few instances where the strain on services during the pandemic impacted on the availability and effectiveness of services, including palliative care provision.

### 3.6 Treatment for COVID‐19

Initially, there was no recommended treatment for COVID‐19^4^; most treatment interventions aimed to relieve the symptoms of the virus. Home‐based treatments included rest, drinking plenty of fluids, taking over the counter pain relief and anti‐inflammatory medicines and easing breathlessness through environmental or postural adjustments. Hospital‐based treatments included intravenous fluids and antibiotics, breathing support with the use of oxygen or ventilation and medication or other treatments to counter the effects of the virus.

In this cohort, the majority of those who died from COVID‐19 were treated with antibiotics (69%, *n* = 112) and/or oxygen (61%, *n* = 99). A smaller proportion (15%, *n* = 25) received mechanical breathing support or ventilation, in line with proportions in the general population (Harrison et al., 2020). Most (76%, *n* = 124) had received treatment in hospital; of these, 9% (*n* = 14) had received some of their treatment in an intensive care unit, high dependency unit or critical care unit.

### 3.7 Recognition of deterioration prior to death

It is vital that indications that a person's health is deteriorating are detected and recognised promptly, and action is taken to escalate care. The New Early Warning Score 2 (NEWS2) (Royal College of Physicians, 2017) is endorsed by NHS England and NHS Improvement as the recognised early warning system for identifying acutely ill and deteriorating patients in hospitals in England. It is also, increasingly, being used in primary care and community settings.

Almost a quarter (23%, *n* = 37) of people who died from COVID‐19 and 12% (*n* = 5) of those who died from other causes had one or more NEWS2 scores recorded, either as a single recording to support decision‐making or as a sequence of recordings to monitor potential deterioration. Some concerns were raised about the absence of the use of tools such as NEWS2 that could have been used to detect acute deterioration, and several recommendations were made about the need for oxygen saturation monitors to be available in care homes.

For some people, however, relying on a monitoring tool needed to go hand‐in‐hand with picking up on the ‘softer’ signs that a person was becoming more unwell, with the need for clearer guidance for families and paid carers about identifying acute deterioration and a reminder to professionals to listen to those who know a person best who may be expressing concern about a person's condition:

Often the subtle signs that are picked up by carers about a deterioration in health are not always identified within the algorithm [used to prioritise calls to NHS111] so may not trigger an alert. COVID‐19 has caused a need to reassess what information is required from individuals contacting the 111 service, especially when the information is being given on behalf of someone who has communication difficulties. There does not appear to be any acknowledgement of level of concern by a carer.

### 3.8 Do not attempt cardio‐pulmonary resuscitation decisions^5^

We reported in the LeDeR programme annual report 2019 that of 1875 deaths of adults reviewed in 2019 for whom data was available about do not attempt cardio‐pulmonary resuscitation (DNACPR) decisions, 72% had such a decision. Reviewers felt that the majority of those (78%) were correctly completed and followed.

Information about DNACPR decisions was available for all our sample population. Of those who died from COVID‐19, 82% had such a decision. Reviewers felt that the majority of these (72%) were correctly completed and followed. Where this was not the case, several people's reviews indicated that frailty or intellectual disabilities were given as a rationale,^6^ or that DNACPR decisions had not adhered to the Mental Capacity Act (MCA) because an assessment of capacity had not been undertaken or next of kin contacted.

Of those who died from other causes, 72% had such a decision. Again, reviewers felt that the majority of these (87%) were correctly completed and followed. None of those who died from causes of death unrelated to COVID‐19 had DNACPR decisions made on the basis of a frailty score or because the person had intellectual disabilities, and none stated that the process had not adhered to the MCA.

### 3.9 Overall assessment of the quality of care

At the end of their review, having considered all the evidence available to them, reviewers are requested to provide an overall assessment of the quality of care provided to the person. The grading is as follows:

1. Care met or exceeded good practice.
2. Care fell short of current good practice in one or more minor areas, but this did not significantly impact on the person's well‐being.
3. Care fell short of expected good practice in one or more significant areas, but this did not significantly impact on the person's well‐being.
4. Care fell short of expected good practice and this significantly impacted on the person's well‐being and/or had the potential to contribute to the cause of death.
5. Care fell far short of expected good practice and this contributed to the cause of death.

**FIGURE 1:**
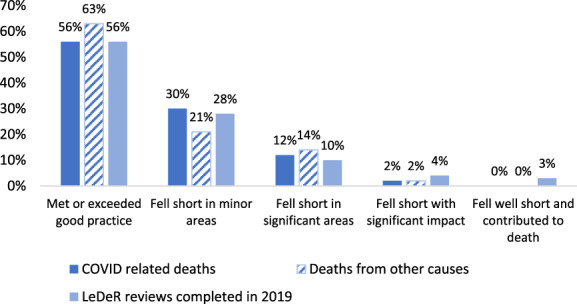
Reviewer assessments of the quality of care provided to adults with intellectual disabilities who died from COVID‐19, those who died from other causes and all LeDeR reviews completed in 2019

Figure 1 presents the reviewers' assessment of the quality of care provided to adults with intellectual disabilities who died from COVID‐19, and those who died from other causes. It also shows the overall quality of care for those whose deaths were reviewed in 2019.

Just over half (56%) of people who died from COVID‐19 and 63% of those who died from other causes received care that reviewers graded as meeting or exceeding good practice (Grade 1). The corresponding proportion of deaths reviewed in 2019 was also 56%.

Care received by 2% of those who died from COVID‐19, and 2% of those who died from other causes, was recorded to have fallen so far short of good practice it had a significant impact on the person's health (Grades 5 and 6). The corresponding proportion of deaths reviewed in 2019 was 7%. This was most frequently due to delays in the diagnosis and treatment of people, a lack of proactive care, the provision of poor‐quality care and deficiencies in monitoring existing health conditions.

### 3.10 Broader impacts of COVID‐19 on the lives of people with intellectual disabilities

A range of broader impacts of the COVID‐19 pandemic were mentioned in completed reviews. These were predominantly in relation to four key issues:

That face‐to‐face visits were restricted and contact with the person by families or professionals was by telephone or video link:

Due to guidelines his mother was unable to visit Neil.^7^ Face to face discussion with professionals may have resolved any concerns from [his mother] and established her understanding of his condition and prognosis.

Delays in the provision of clinical care, particularly hospital admissions for both routine and emergency care:

Ewan had had a scan booked for his pancreas …but this was cancelled due to the COVID risk.

Changes to the availability and skillset of paid carers, particularly due to sick leave within the organisation:

The COVID pandemic and the demands on the NHS (including the redeployment of staff) impacted on the availability and effectiveness of services to support Ms Brown.

The impact of COVID‐19 on end‐of‐life care and funeral arrangements:

The inclusion of COVID‐19 on Habibah's death certificate caused her family problems with arranging her burial as most of the mosques they approached refused to do it. Eventually a mosque that is far away from where they live agreed to do it.

Other broader impacts of the COVID‐19 pandemic were in relation to the closure of day services, delays to existing plans, the isolation of people with intellectual disabilities and an increase in clinical responsibilities for family carers.

### 3.11 Recommendations from reviews of deaths

Seventy‐six recommendations were made by LeDeR reviewers in relation to COVID‐19. We have described these as Findings because they are the outcome of analysis of the findings of completed reviews, not the authors own views.

A cluster of the recommendations focused on the identification of illness and recognition of deterioration. Among these, recommendations included the use of specific deterioration tools such as NEWS2; paying particular attention to the concerns of families and paid carers about subtle signs that a person may be unwell; and the use of pulse oximeters in community settings.

Other clusters of recommendations were in relation to the need for enhanced availability of specialist intellectual disability nurses in hospital settings; the use of reasonable adjustments to enable people with intellectual disabilities to have a familiar person with them in hospital; safe hospital discharge; the availability and use of PPE; COVID‐19 testing for staff and residents; the need for bereavement support as appropriate; and the need to plan proactively to ensure services had sufficiently robust plans for staffing and equipment in case of high demand.

## 4 CONCLUSIONS

This report describes the circumstances leading to death of a sample of 163 adults with intellectual disabilities whose deaths were notified to the English LeDeR programme and who died from suspected or confirmed COVID‐19. To our knowledge, this is the largest study yet about the specific circumstances of people with intellectual disabilities who died with COVID‐19 in the first wave of the pandemic in England. Its strength is that all deaths have been reviewed using the standardised LeDeR programme framework, so the data is reasonably consistent. In addition, a small comparator group of people who died from other causes allows some comparison in relation to the provision of care. There are, however, also limitations. This is a study of 163 deaths so the numbers relating to some variables, particularly if disaggregated by demographic or other factors, are small. As such, regional differences from within England have not been reported, nor have deaths from specific genetic causes such as Down's syndrome. Deaths were reviewed at a time of considerable pressure on the health and care sectors, which may have been reflected in the quality or completeness of some reviews. In addition, we must bear in mind that the LeDeR programme was established as a service improvement initiative, not a research study.

Nevertheless, from the findings, a number of conclusions can be drawn about deaths of people with intellectual disabilities in England from COVID‐19 that could contribute to service improvements.

First, there was a striking difference in age at death people in the general population who died with COVID‐19 compared with people with intellectual disabilities. This is likely to be reflective of the lower average age at death of people with intellectual disabilities generally (Glover et al., 2017; Lauer & McCallion, 2015; Trollor et al., 2017), but it suggests that risk of death from COVID‐19 is not limited to people in the oldest age groups and were age thresholds for shielding people from COVID‐19 or for vaccinations against COVID‐19 be introduced, they would disproportionately disadvantage people with intellectual disabilities.

Second, none of the people with intellectual disabilities who died with COVID‐19 were thought to have reported an altered sense of smell or taste, suggesting that this symptom is difficult to identify in people with intellectual disabilities so should not be relied on. Vlaskamp and Cuppen‐Fonteine (2007) suggest that a range of barriers exist in assessing sensory functioning in people with intellectual disabilities. People may have specific, idiosyncratic thresholds for stimuli; they may be unable to focus on sensory stimuli sufficiently long to communicate about it; may not be fluent verbally and thus unable to report themselves; and their behavioural responses may be difficult to interpret and be subject to the assessment of another person.

Third, it would seem appropriate to target public health and preventative measures at people with intellectual disabilities who have respiratory disease, hypertension or obesity. Although these have been identified as being associated with a greater risk of death from COVID‐19, the pandemic has exposed concerns about the general health status of some members of the population and the need to reduce existing health inequalities (Bibby et al., 2020; Douglas et al., 2020).

Fourth, the study has identified concerns about recognising deterioration in people with intellectual disabilities in community settings. This has been raised as an issue of concern in the support of people with intellectual disabilities in previous years (Heslop et al., 2020) and is not specific just to COVID‐19, but the pandemic has heightened awareness about the scale of the problem and the need for an urgent resolution. Enhanced ‘safety netting’ (providing information about what to expect in the course of an illness and how to access medical help if there are any concerns), the provision of specific advice about recognising signs of deterioration and the introduction in community settings of tools (and the specific equipment required for these, such as oxygen saturation monitors) used to detect acute deterioration in a person's health would all be appropriate.

Fifth, access to healthcare was problematic for some people who died with COVID‐19. Although it has been documented that there have been problems for people in general accessing NHS111 (Park et al., 2020; Phillips, 2020) and COVID‐19 tests (Wise, 2020), people with intellectual disabilities also had difficulties accessing specialist teams who could support them to do so. In some areas, specialist intellectual disability nurses were redeployed to care for COVID‐19 patients, or moved to online consultations, without the full impact of this being considered. Ensuring the availability of specialist intellectual disability teams to support people with intellectual disabilities, their families and paid carers would improve the ways in which services are delivered to meet the needs of people with intellectual disabilities.

We need a clear understanding of the COVID‐19‐related factors associated with deaths of people with intellectual disability. Some, including symptoms of COVID‐19 and age at death, are different in people with intellectual disabilities compared with the general population. Although the overall quality of care was rated similar to other deaths of people with intellectual disabilities, concerns were raised relating to recognising acute deterioration and DNACPR decisions, indicating the need for service improvements in these particular areas. Translating the findings from this and other studies into robust ways of preventing COVID‐19 in people with intellectual disabilities and supporting those who come into contact with the disease must be prioritised to prevent further health inequalities in this population.

In this small study, we have focused on some of the health and care‐related experiences of people with intellectual disabilities who died from COVID‐19 and offered recommendations for future service improvement. However, many social determinants of health (Dahlgren & Whitehead, 2006), including poverty and aspects of the physical environment can have a considerable effect on COVID‐19 outcomes (Abrams & Szelfler, 2020) and are under‐recognised, including in people with intellectual disabilities. A broader focus of future research is, therefore, needed on the social determinants of health, which have a significant influence on a person's chances to die from COVID‐19.

Finally, the COVID‐19 pandemic has shone a light on some of the existing disparities in society (Bambra et al., 2020; The Independent Scientific Advisory Group for Emergencies (SAGE), 2020), including between people with and without intellectual disabilities (Schormans et al., 2021). As Schormans et al. (2021) argue, as we move out of the pandemic, we should not return to ‘normal’, but must, as a priority, address long‐standing deficiencies in the care of people with intellectual disabilities and their families that the COVID‐19 pandemic has illuminated.

## Data Availability

Author elects to not share data.

## Footnotes

^1^
Further information about the programme is available at www.bristol.ac.uk/sps/leder

^2^

Available on request from the author.

^3^
See: http://www.bristol.ac.uk/sps/leder/about/detailed-review-process/

^4^
At the time of writing, Remdesivir, corticosteroids, tocilizumab and sarilumab have since been recommended as treatments for COVID‐19 in some patients. See: https://www.england.nhs.uk/coronavirus/wp-content/uploads/sites/52/2020/07/C0654-uk-interim-clinical-commissioning-policy-remdesivir-for-patients-hospitalised-with-COVID-19-adults-and-c.pdfand https://www.nice.org.uk/guidance/ng159/resources/covid19-prescribing-briefing-corticosteroids-pdf-8839913581

^5^
Cardio‐pulmonary resuscitation is when a person receives chest compressions and artificial breaths to help pump blood around their body when their heart has stopped. A decision not to attempt cardio‐pulmonary resuscitation is made and recorded in advance when it would not be in the best interests of the person because they are near the end of their life or the procedure would be unlikely to be successful.

^6^
On 25 March 2020, NICE clarified that the Clinical Frailty Scale should not be used for younger people, people with stable long‐term disabilities, learning [intellectual] disabilities or autism.

^7^
All names have been anonymised to protect confidentiality.
